# Special Issue “Artemisinin (Qinghaosu): Commemorative Issue in Honor of Professor Youyou Tu on the Occasion of Her 80th Anniversary” in International *Molecules* Published in 2010

**DOI:** 10.3390/molecules26020279

**Published:** 2021-01-08

**Authors:** Yan He, Shu-Kun Lin

**Affiliations:** 1 Songshan Lake Materials Laboratory, Dongguan 523808, Guangdong, China; heyan@sslab.org.cn; 2 MDPI, St. Alban-Anlage 66, 4052 Basel, Switzerland

It has been more than 10 years since we published the Special Issue “Artemisinin (Qinghaosu): commemorative issue in honor of Professor Youyou Tu on the occasion of her 80th anniversary” (Abbreviated as “the Artemisinins Special Issue”) [1].

Artemisinin and its derivatives are a group of antimalarial agents. Artemisinin was first discovered in China in the 1970s under the “523” research project with Tu Youyou as the leader of a team searching for a new antimarial drug from Chinese herbs [2]. Almost 40 years after the discovery and 5 years after the publication of the Special Issue [1], Tu Youyou was awarded the 2015 Nobel Prize in Physiology or Medicine “for her discoveries concerning a novel therapy against Malaria” [3]. Tu Youyou was the first native Chinese Nobel laureate in Medicine or Physiology. 

*Molecules* has a journal section focusing on small-molecule drugs. As the publisher of *Molecules*, Shu-Kun Lin planned to run this Special Issue and he visited Tu Youyou in December 2008. Yan He was the Managing Editor of *Molecules*, and contacted experts to be the guest editor, Dr. Geoff Brown; together with Dr. Geoff Brown, we organized 12 papers finally published after peer review.

In order to recognize and reward excellence and innovation in the area of natural products and medicinal chemistry, *Molecules* started the Tu Youyou Award [4] in 2016, which has now run for consecutively four years.

Looking over the past 25 years in which *Molecules* has served the scientists in the area of chemistry, it has published many outstanding works and “the Artemisinins Special Issue” is just one of them.
